# Advanced Delivery System of Polyphenols for Effective Cancer Prevention and Therapy

**DOI:** 10.3390/antiox12051048

**Published:** 2023-05-05

**Authors:** Koung Hee Kim, Mi-Ran Ki, Ki Ha Min, Seung Pil Pack

**Affiliations:** 1 Department of Biotechnology and Bioinformatics, Korea University, Sejong 30019, Republic of Korea; wood1018@korea.ac.kr; 2 Institute of Industrial Technology, Korea University, Sejong 30019, Republic of Korea; allheart@korea.ac.kr (M.-R.K.); alsrlgk@korea.ac.kr (K.H.M.)

**Keywords:** polyphenol, antioxidant, anti-inflammatory, anti-cancer, drug delivery system

## Abstract

Polyphenols from plants such as fruits and vegetables are phytochemicals with physiological and pharmacological activity as potential drugs to modulate oxidative stress and inflammation associated with cardiovascular disease, chronic disease, and cancer. However, due to the limited water solubility and bioavailability of many natural compounds, their pharmacological applications have been limited. Researchers have made progress in the development of nano- and micro-carriers that can address these issues and facilitate effective drug delivery. The currently developed drug delivery systems maximize the fundamental effects in various aspects such as absorption rate, stability, cellular absorption, and bioactivity of polyphenols. This review focuses on the antioxidant and anti-inflammatory effects of polyphenols enhanced by the introduction of drug delivery systems, and ultimately discusses the inhibition of cancer cell proliferation, growth, and angiogenesis.

## 1. Introduction

Cancerous or malignant tumors are characterized by uncontrolled cell proliferation and are harmful to human health. Malignant cells do not stay at one spot and migrate to surrounding tissues or metastasize to more distant sites via the circulatory system [1]. This is caused by factors such as oxidative stress and inflammation, which can damage cell and tissue, leading to tumorigenesis or tumor aggravation [2,3]. Chronic inflammation causes persistent damage to cell membranes, proteins, and DNA, supporting cancer development by promoting cell proliferation, DNA mutation, and the formation of blood vessels that feed tumors [4,5]. Polyphenols can inhibit cancer progression at various stages. A balance between inhibitors and factors that cause abnormal cellular changes reduces the likelihood of cancer development during the early phase [6]. However, polyphenols can act as powerful anticancer agents even in cases where a cancer has progressed.

**Figure 1:**
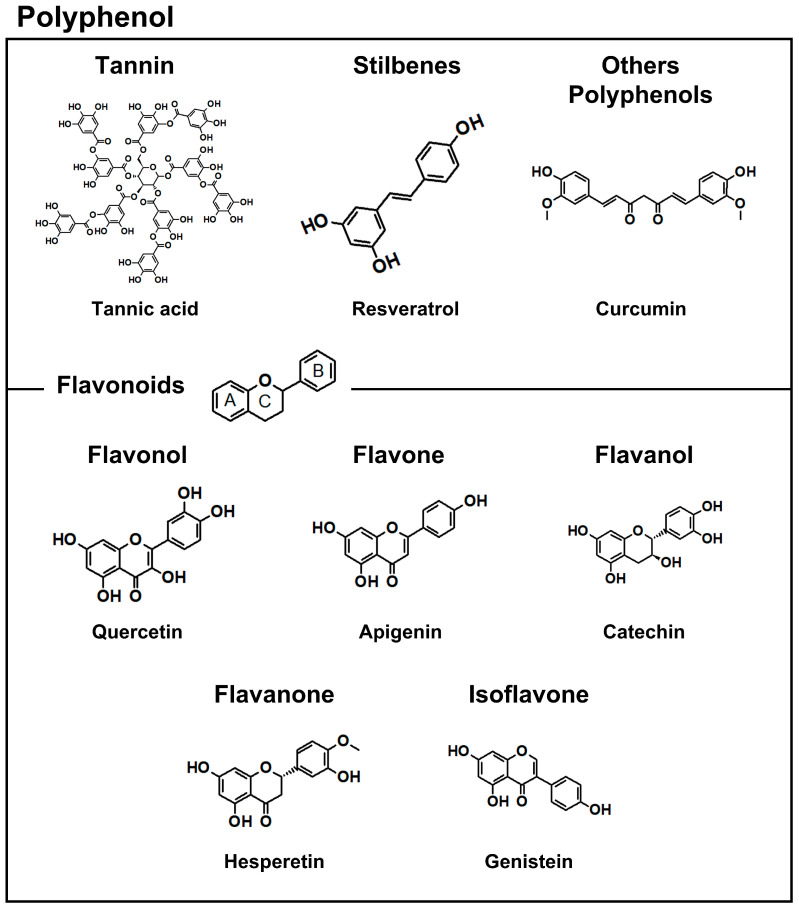
Classification of polyphenols and structure of representative substances.

Recently, interest in the therapeutic application of polyphenols has increased, since these phytochemicals possess antioxidant, anti-inflammatory, anticancer, cardiovascular-protective, and anti-obesity properties [7,8]. They are found in many natural sources, such as woods, vegetables, fruits, seeds, and roots. Polyphenols generally contain an aromatic ring with one or more hydroxyl groups. According to the functional groups, polyphenols can be classified as phenolic acid, flavonoid, tannin, etc. (Figure 1) [9]. Various polyphenols are also being studied in combination with other anticancer drugs to enhance their effects.

**Figure 2:**
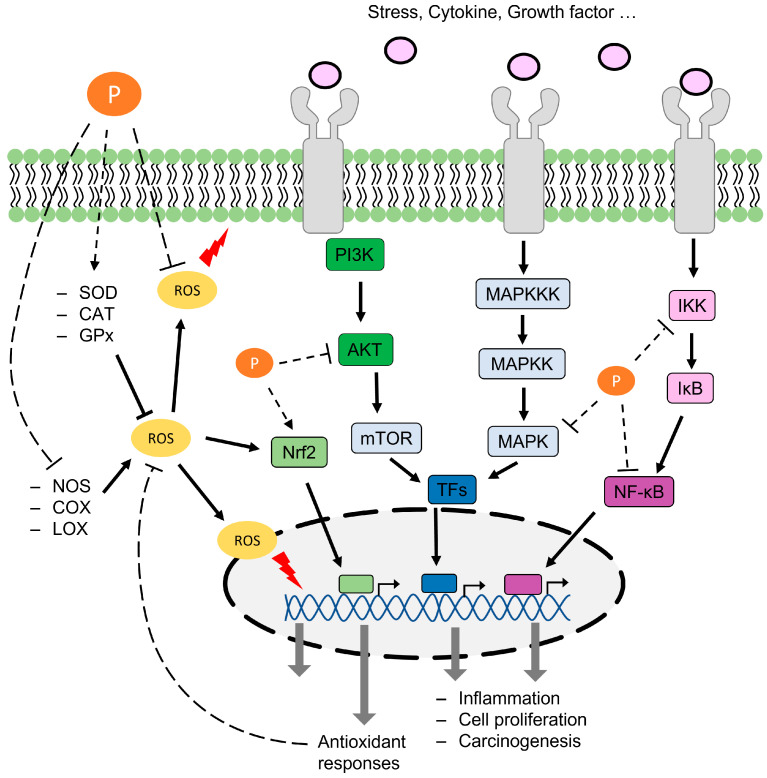
Anticancer effects of polyphenols involve antioxidant and anti-inflammatory effects [32,33,34,35,36,37,38,39,40,41,42]. P (orange): polyphenol; TFs (blue): transcriptional factors.

Polyphenols exert antioxidant effects by scavenging free radicals, chelating metal ions, and modulating the activity of enzymes involved in the production or removal of ROS. Polyphenols contain a benzene ring with multiple hydroxyl groups, and this benzene ring can provide electrons or hydrogen ions to free radicals, thus contributing to the elimination of oxidative stress [10]. Moreover, the phenol group of polyphenols can interact with and chelate metal ions. Among the metal ions, Fe2+ ions react with peroxides via the Fenton reaction to produce hydroxyl radicals [11]. Another mechanism involves the interaction of polyphenols with enzymes responsible for regulating ROS, such as peroxidase, nitric oxide synthase (NOS), cyclooxygenase (COX), lipoxygenase (LOX), superoxide dismutase (SOD), catalase (CAT), and glutathione peroxidase (GPx) [12,13,14,15]. Inflammation is a complex immune response to bacterial infections, tissue damage from physical trauma or chemicals, and allergic reactions that recruit immune cells to produce a variety of signaling molecules, including cytokines, ROS, and reactive nitrogen species (RNS) [16]. Chronic inflammation is associated with several diseases, including cancer [17], obesity [18], allergies [19], and neurological disorders [20]. Polyphenols have been studied for their ability to regulate inflammation by modulating transcription factor activity [21,22,23,24], protein function [25,26,27,28], and cell signaling [29,30,31], making them potential candidates for use as anti-inflammatory agents. The antioxidant and anti-inflammatory effects of polyphenols involved in cancer are illustrated in Figure 2.

However, the therapeutic applications of phenolic compounds have several limitations. These compounds have poor water solubility and can be easily degraded under the influence of environmental factors, such as pH, temperature, and light, resulting in low absorption, poor bioavailability, etc. [43]. Moreover, rapid clearance and high metabolization reduce their therapeutic efficacy and compromise their biological effects [44]. To overcome these problems, drug delivery systems (DDSs) using biocompatible materials with various properties are being developed [45].

DDSs are designed for the targeted delivery of optimal doses of drugs and their controlled release [46]. DDSs for polyphenols generally aim to increase their bioavailability and bioaccessibility by improving stability and solubility [47]. Moreover, high loading and selective delivery maximize the bioactivity of polyphenols by preventing drug overdose and the resulting side effects [48]. Recently, several studies have tried to maximize the anticancer effects of polyphenols using DDSs, such as nanoparticles, including liposomes, phytosomes, natural and synthetic polymers, lipid nanoparticles, micelles, protein nanoparticles, and inorganic complexes.

This review discusses the bioactivities of polyphenols and describes their ameliorative effects on oxidative stress and inflammation in cancer. Our review also describes recent studies aimed at improving the anticancer effects of polyphenols using the DDSs.

## 2. Enhancement of Polyphenol Anticancer Effects Using DDSs

**Figure 3:**
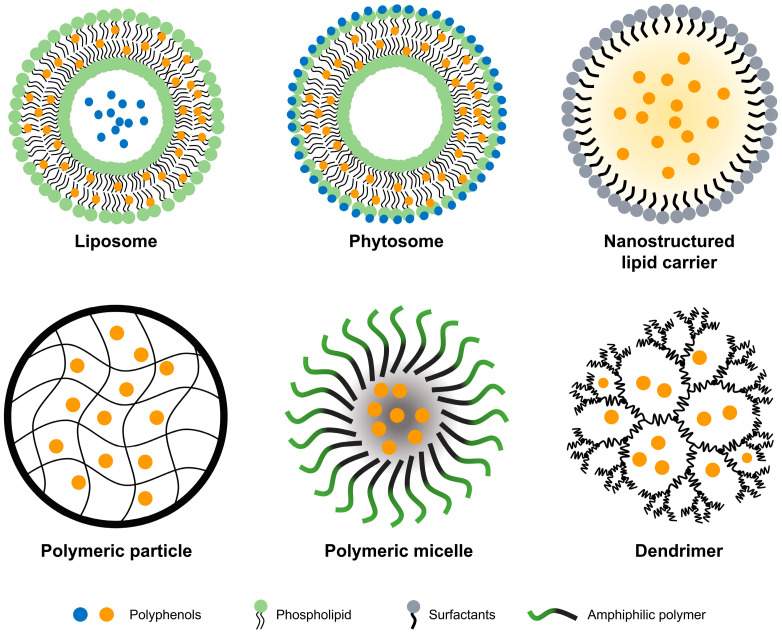
Representative drug delivery systems for polyphenol.

Polyphenols have great potential for preventing and treating cancer. However, their poor solubility and stability limit their potential use as therapeutics. The development of delivery systems capable of overcoming critical drawbacks has been emphasized to prevent the precipitation, rapid degradation, and clearance of bioactive compounds and improve their bioavailability [49,50]. Nanoformulation enables high loading efficiency, drug protection, and specific delivery by exploiting the differences between cancer cells and normal cells within the tissue [51,52]. This section reviews the properties of several polyphenol-loaded nanoparticles (Figure 3) and their anticancer effects.

### 2.1. Lipid-Based System

Lipid-based formulations are most commonly used for entrapping polyphenols with low water solubility. Lipid nanoparticles can improve biocompatibility, cellular penetration, and drug stability for effective drug delivery. Widely used formulations include liposomes, phytosomes, emulsions, and nanoparticles [53].

#### 2.1.1. Liposomes and Phytosomes

Liposomes are the most recognized nanoparticles for drug delivery. Their primary material, phospholipids, has a hydrophilic head and a hydrophobic tail, which allows them to form nanoparticles with a bilayer structure similar to a cell membrane. The liposome core is hydrophilic, therefore it can be loaded with water-soluble substances, while the membrane is hydrophobic, protecting the drug from the external environment [54]. Jhaveri et al. developed polyethylene glycol (PEG)ylated liposomes loaded with resveratrol and showed that these liposomes can cross the blood–brain barrier (BBB) [55]. The authors also modified the PEGylated resveratrol–liposome (RES–LP) particles with parts of transferrin (Tf) for targeting Tf receptors on glioblastoma. The particles exhibited homogeneous size (182.3 ± 12.1 nm for RES–LP without Tf and 211.2 ± 0.8 nm for RES–LP with Tf (Tf–RES–LP)) and encapsulated approximately 70–75% of all resveratrol. Compared to normal cells, liposomes with Tf showed a 2.3-fold higher interaction with cancer cells expressing Tf receptors. The viability of U-87 MG human glioblastoma cells was 54.2 ± 3.8% at 24 h after incubation with Tf–RES–LP particles and 66.9 ± 6.2% at 4 h, whereas the viability was 80.4 ± 9.6% when cells were incubated with free resveratrol and 83.0 ± 2.9% when incubated with RES–LP. The authors also showed that resveratrol and resveratrol-loaded liposomes induced apoptosis by activating caspase 3/7. Moreover, these particles efficiently decreased tumor growth in mice. Mice treated with Tf–RES–LP exhibited lower tumor volume compared to those treated with free resveratrol, confirming that liposomal formulation of resveratrol with Tf on the surface improves the anticancer effect of this polyphenol. Another study used peptide liposomes to form resveratrol delivery carriers for targeting breast cancer [56]. The average size of the peptide liposome-loaded resveratrol (PSL@RES) was approximately 140 nm, and these particles exhibited stability for three months at 4 °C. The resveratrol release rate from PSL@RES was 96% at pH 5.5, which was higher than that at pH 6.8. The authors suggested that peptide liposomes reduce resveratrol release in blood circulation at neutral pH. Free resveratrol and peptide liposomes were less toxic, with normal cells exhibiting over 80% viability when incubated with these particles, whereas the viability of MCF-7 human breast cancer cells treated with PSL@RES was 23%, which was slightly lower than that of cells exposed to free resveratrol. Moreover, tumor growth in mice was efficiently reduced upon treatment with PSL@RES, and this effect was greater than that of free resveratrol at the same concentration. Mechanistically, the authors demonstrated that PSL@RES involves p53, Bax, Bcl-2, and caspase signaling, thus inducing MCF-7 apoptosis. This suggests that peptide liposomes are an excellent carrier for resveratrol and can reduce the side effects of high resveratrol concentration.

Co-delivery is the delivery of two substances with different functions together to achieve synergistic effects. Zhou et al. reported the co-delivery of gefitinib (GFT) and curcumin loaded in liposomes with PEGylated phospholipids and soybean phosphatidylcholine (PC) [57]. GFT is an inhibitor of the epidermal growth factor receptor (EGFR) tyrosine kinase activity in diverse tumors. Curcumin, one of the most studied polyphenols, exerts antioxidant and antitumor effects when used alone or synergistically when used with other drugs for multidrug-resistant cancer. These liposomes exhibited a particle size of approximately 110–130 nm and a high encapsulation efficiency (EE) (>80%) when used with single or both drugs. Dual-drug liposomes showed sustained release in phosphate-buffered saline (PBS) and higher cellular uptake than free forms and single-drug liposomes. Moreover, the viability of PC-9 cancer cells was slightly lower when incubated with GFT/curcumin liposomes compared to that of cells exposed to free forms of drugs. Interestingly, the viability of GFT-resistant H1975 cells was dramatically different. Co-delivery using dual-drug-loaded liposomes improved the inhibitory effects on GFT-resistance cancer cells compared to co-treatment with single-drug-containing liposomes. Similar results were observed in vivo. The tumor growth in mice was reduced from 7.6 (5.5)-fold and 6.0 (4.5)-fold to three-fold when treated with free GFT (and with free curcumin), GFT liposomes (and with curcumin liposomes), and GFT/curcumin liposomes, respectively. Thus, co-delivery of curcumin using the liposomal formulation can improve and boost its anticancer effects on multi-drug-resistant cancers by increasing its cellular uptake and inhibiting cell growth.

Phytosomes are similar to liposomes, since they have phospholipids as the primary constituents. However, in phytosomes, the phospholipids and polyphenols form a complex based on hydrogen-bonding [58,59]. This interaction increases the bioavailability and stability of the polyphenols in the body and facilitates cellular uptake [60]. Komeil et al. used phosphatidylcholine (PC) for phytosome formulation of genistein, an isoflavone from soy, for targeting hepatocellular carcinoma (HCC) [61]. They used three different types of PCs (pure, with medium-chain triglycerides (MCT), and with long-chain triglycerides (LCT)) to increase the solubility and bioavailability. The particle size of phytosome-loaded genistein was approximately 150–230 nm, and the EE was over 95%. When used with this formulation, genistein was significantly accumulated in the liver and its metabolism was significantly decreased compared to cases when free genistein and formulation with MCT were used because of increased lymphatic uptake and strong complex. Although PC formulation significantly increased the cellular uptake, cytotoxicity of free genistein was higher than that of genistein-phytosomes after 24 h of treatment but lower after 48 h. The authors showed that -OH groups of genistein interact with phosphatidylcholine present in phytosomes, resulting in reduced cytotoxicity; however, after 48 h, cells recover due to hydrogen-bonding breakdown. Increased levels of aspartate aminotransferase (AST) and alanine aminotransferase (ALT) in the serum reflect hepatotoxicity. Genistein-phytosomes with pure PC reduced the levels of AST by 30% and ALT by 10%, thus protecting the liver from damage and toxicity. Moreover, genistein-phytosomes increased the levels of caspase 8, resulting in caspase 3 activation (caspase-dependent apoptosis effector) and release of mitochondrial oxidoreductase (caspase-independent apoptosis activator). Furthermore, they suppressed the expression of matrix metalloproteinases and vascular endothelial growth factor-A (VEGFA), molecules that are related to angiogenesis and tumor invasion. Finally, histopathological improvements in rats suggested that phytosomes with pure PC or LCT can enhance the anticancer effects of genistein by reducing damage to the liver and tumorigenesis. Thymoquinone (TQ) present in black seed oil is used for respiratory conditions and to boost immunity. TQ can be formulated using a soy-phospholipid-based phytosome [62]. Using a Box-Behnken design tool, a previous study optimized the generation protocol, producing particles with a size of 45.59 nm. TQ has poor water solubility and bioavailability. TQ release from TQ-phytosomes was over 95%, which was higher than that from the TQ suspension (49%) because of the structural stability of the formulation and the sustained release ability from the core to the surface. When treating A549 human lung cancer cells, the IC50 value of TQ-phytosomes was significantly lower than that of free TQ, which is similar to the effect of blank phytosomes on EA.hu925 normal cells. The anticancer effects of TQ-phytosome were due to its ability to induce cell cycle arrest by promoting caspase 3 expression and ROS generation in cancer cells. Thus, the phytosome formulation induced apoptosis and necrosis in human lung cancer cells. 

Another study using a soy-phospholipid-based phytosome developed curcumin-soybean phosphatidylcholine (CUR-SPC) phytosomes with PEGylation and folate (FA) modification, a ligand of FA receptor overexpressed in certain cancers [63]. These modifications enhanced the particle stability, retention time, and tumor cellular uptake. CUR-SPC was found to be soluble in toluene, and compared to a simple mix, it showed different characteristics, suggesting that the interaction between polyphenols and the polar head of SPC is important for solubilization. The phytosome had a uniform size of about 185.3 nm and a negative surface charge, both of which were advantageous for enhanced penetration and retention (EPR) and circulation in the blood. The loading capacity (LC) of PEG-modified curcumin-phytosome with FA was 24.3%, which was similar to or slightly lower than that of non-modified particles. The release of the formulation was 20% at pH 7.4, which is similar to the pH of blood but increased to about 50% at pH 5.5, which is similar to the pH of the tumor microenvironment. Moreover, phytosome formulation significantly enhanced curcumin uptake by HeLa cells and reduced the cytotoxicity associated with the free drug. The in vivo test showed that the particles gathered around the cancer cells in real time and accumulated more within the tumor tissue compared to the normal tissue. Thus, PEG protection enhances stability, and FA receptor-mediated specificity promotes endocytosis of these phytosomes. *Moringa oleifera* leaf polyphenols (Mopp) exert anticancer effects against breast cancer; however, due to their hydrophilicity, these polyphenols exhibit low cell permeability and are degraded in the gastrointestinal tract. Wanjiru et al. entrapped Mopp using phytosomes (MoP) to overcome the issues [64]. The EE of MoP was 90.32%, which was calculated by evaluating the total phenolic compounds present in phytosomes against the same concentration of Mopp. The particles were homogenous with an average size of 296 nm and a high negative charge on the surface (−40.1 mV). In vitro release profile of MoP was boosted early and sustained after 8 h due to the difference in polyphenol positions. The phytosome formulation significantly enhanced the stability and protected the polyphenols from environmental factors, such as digestive enzymes and gastric fluid, thus increasing their bioavailability more than two-fold. The cytotoxicity of MoP against cancer cells was four-fold higher than that of the Mopp extract. Moreover, MoP dose-dependently inhibited the proliferation of 4T1 cancer cells, and this effect of MoP was more efficient than that of doxorubicin (DOX) at a high concentration (100 µg/mL). These results showed that limitations associated with the use of Mopp can be improved using the Mopp-phytosome, and these phytosomes are efficient in breast cancer treatment.

#### 2.1.2. Nanostructured Lipid Carrier

Researchers are trying to develop DDSs using biocompatible solid or liquid lipids and amphiphilic materials such as phospholipids to create nanoemulsions or nanoparticles. Nanostructured lipid particles (NLPs) have several advantages, such as controlled release, targeted delivery through surface modification, high stability, and high EE [65]. Kanwal et al. developed a self-nanoemulsion with the absorption enhancer sodium N-[8-(2-hydroxybenzoyl) amino]caprylate (SNAC) for curcumin formulation [66]. The authors used oil, a surfactant, and a co-surfactant for preparing a curcumin-loaded self-nanoemulsifying drug delivery system (CUR-SNEDDS), and selected cinnamon oil, Tween 80, and PEG 200 based on curcumin solubility and emulsification abilities. The size of the emulsion particles increased from 22 nm to 106 nm when curcumin was added, suggesting that the drug was solubilized by the surfactant and co-surfactant and accumulated in the emulsion. The addition of SNAC to CUR-SNEDDS (SC-CUR-SNEDDS) reduced the release rate in acidic and alkaline conditions. In Caco-2 human colon cancer cells, CUR-SNEDDS and SC-CUR-SNEDDS showed a cytotoxicity of 29.45% and 57.28%, respectively, when low concentrations of curcumin were used and no effect of free curcumin was observed. The cytotoxicity was 88.83% and 98.49% for CUR-SNEDDS and SC-CUR-SNEDDS, respectively, when the highest concentration of curcumin was used compared to 67.09% of free curcumin. The authors orally treated the mice with curcumin-loaded SNEDDS and checked the pharmacokinetic parameters. Compared to the curcumin suspension, the maximum plasma concentration (C_max_) of SC-CUR-SNEDDS was five-fold higher, while the AUC was 4.5-fold higher. Furthermore, SC-CUR-SNEDDS exhibited a longer retention time compared to the curcumin suspension. This study demonstrated that SNEDDS with a SNAC absorption enhancer for curcumin is an excellent oral delivery system for improving oral bioavailability and cell absorption.

Radbeh et al. used *Cornus mas* extract (CME) with cubosome, a nano-sized cubic liquid crystallized using glyceryl monooleate, Poloxamer^®^ 407, and Eudragit^®^ S100 [67]. Cubosomes have aqueous channels that dynamically open and close, and a vesicular liposome-like structure that functions as a carrier for diverse bioactive compounds. The CME-loaded nanocubosomes (CME-NCs) showed a particle size of 22.75 nm, which is advantageous for cell penetration, while the EE and LC were found to be 95.14% and 9.51%, respectively. CME abundantly contains anthocyanin, a powerful antioxidant; however, anthocyanin can be easily degraded and inactivated in the gastrointestinal tract. While evaluating the anthocyanin stability in CME-NCs and free CME, the authors found that the 2,2-diphenyl-1-picrylhydrazyl (DPPH) scavenging activities were higher in CME-NCs, with a value of 92%, compared to free CME, which only showed a value of 75%. However, both CME-NCs and free CME exhibited a decrease in DPPH scavenging activity over time. In addition, the authors confirmed that cubosomes significantly enhance the stability of phenolic compounds. In the HT-29 human colon carcinoma cell line, cell viability showed a correlation with CME concentration, and reduction in viability was more pronounced when cells were incubated with CME-NCs compared to free CME. Moreover, CME-NCs increased the DNA fragmentation and the proportion of cells in the G0-G1 phase, thus inducing apoptosis rather than necrosis in HP-29 cells. The authors also demonstrated that nanocubosomes with Eudragit^®^ S100 have a higher EE and sustained release properties, and these nanocubosomes can stabilize polyphenols present in CME and inhibit colon cancer progression.

Hajipour et al. improved the anticancer effects of EGCG using the NLP-modified arginyl-glycyl-aspartic acid (RGD) [68]. RGD is a common part of diverse adhesion molecules and is recognized by integrin receptors that are overexpressed in various cancers. NLP displaying RGD can be taken up by cancer cells via receptor-mediated endocytosis. The nanoparticle exhibited a monodispersed size of 85 nm, thus enabling the particles to avoid renal clearance and achieve rapid phagocytic uptake. Moreover, the particle size remained stable and the high EE (80%) was maintained after long-term storage. The cytotoxicity of EGCG-loaded RGD-NLP was higher than that of free EGCG against MDA-MB-231 human breast cancer cells. Co-treatment with DOX and EGCG-loaded RGD-NLP showed significantly high cytotoxicity compared to DOX alone at a low concentration. Low doses of DOX are advantageous to prevent adverse effects on normal cells. RGD modification significantly increased the cellular uptake of EGCG. MDA-MB-231 cells treated with the nanoparticles and DOX underwent cell cycle arrest and apoptosis. These results suggest that RSD-modified NLP enhanced the anticancer effects of EGCG, and when co-treated with DOX, it further boosted the biological activities of the compound.

In another study, researchers encapsulated apigenin (APG) using hyaluronic acid-decorated NLP for targeting lung cancer [69]. APG is a potent inhibitor of Nrf2, a transcription factor involved in non-small-cell lung cancer drug resistance. APG-loaded NLP with hyaluronic acid (HA-APG-NLP) had a particle size of 88 nm and a positive surface charge that can interact with cancer cells having a negative surface charge. The release was slightly lower at the neutral pH than in the acidic solution. The cytotoxicity of HA-APG-NLP against A549 lung cancer cells was approximately three-fold lower than that of APG alone; however, no toxicity to normal cells was observed. Hyaluronic acid, a ligand of CD-44 receptors, enhanced the cellular uptake of NLP in A549 cells. Treatment with HA-ALP-NLP increased the number of apoptotic cells, and co-treatment with docetaxel (DTX), an anticancer drug, was more effective than free APG and/or DTX treatment. These results showed that HA-decorated NLP can efficiently formulate APG to target Nrf2-overexpressing DTX-resistant lung cancer.

The lipid-based systems mentioned above are summarized in Table 1 and Table 2.

### 2.2. Polymer-Based System

Polymers have different structures, charges, thermal stability, and pH sensitivity that can be chosen depending on the drug or target. Natural and synthetic polymers are available as nanoparticles, films, meshes, and hydrogels for various applications. Polymer nanoparticles can be loaded with hydrophilic or hydrophobic polyphenols, and their size and surface area characteristics allow for effective targeted delivery [70].

Chitosan is one of the natural polymers that is generally used for drug delivery. FA is a water-soluble B vitamin whose receptors are overexpressed on the surface of cancer cells and are often targeted for drug delivery [71]. Lazer et al. used the chitosan-FA conjugate to deliver the flavonoid hesperetin to cancer cells [72]. The chitosan-FA conjugate nanoparticle with hesperetin (CFH NPs) had a particle size of 457 nm and was permeable to cancer cells due to the EPR effect. The particles had a high EE of 98% and their cellular uptake was three-fold higher compared to that of non-FA conjugated nanoparticles. The hesperetin-loaded conjugate used hydrogen bonding, and there was a cross-linking between sodium tripolyphosphate and chitosan. CFH NPs exhibited sustained release of about 45% during 72 h in diverse pH solutions, which improved the stability, reduced the side effects to normal cells, and delivered more of the drug to cancer cells. A significant effect of CFH NPs was observed on cell growth inhibition, since these particles target FA receptors that are abundantly expressed on cancer cells. Thus, these particles were taken up by receptor-mediated endocytosis, which prevented the pump-mediated efflux from cancer cells. Additionally, CHE NPs induced apoptosis in cancer cells by regulating the Bax and Bcl-2 antagonist of cell death (BAD) signaling. Compared to hesperetin alone, the nanoparticles were more efficient for targeting cancer.

Sunoqrot et al. used Eudragit S100 synthetic polymer for quercetin formulation [73]. Hydrogen bonding between Eudragit S100 and quercetin led to the formation of a colloidal complex, with a size of 66.8 nm and EE of 41.8%. This polymer is used for enteric coating in pharmaceutical industries. The in vitro release of particles was dramatically controlled, and quercetin was not released in acidic pH (simulated gastric fluid (pH 1.2) and acetate buffer (pH 4.5)) while the release was almost 91% at pH 7.2. This could be explained by the fact that as pH increases, the ionization of polymers is also increased, resulting in polymer swelling and matrix disruption. The colloidal complex of quercetin was cytotoxic to CT26 colon cancer cells with an IC50 of 0.8 µM, while the IC50 of free quercetin was 65.1 µM. Thus, Eudragit polymer-based complex improved quercetin solubility and pH-sensitive release and exhibited highly efficient anti-cancer effects. Thongnopkoon et al. compared the performance of hydroxypropyl methylcellulose 15LV (HPMC15LV) and polyvinylpyrrolidone-K30 (PVP-K30) for preparing curcumin composites by spray drying [74]. When prepared at different concentrations, all particles were smaller than 10 µm, but the composite containing HPMC15LV showed higher curcumin encapsulation efficiency than the PVP-K30 composite. The composite containing HPMC15LV also showed higher solubility in aqueous solutions, which the authors attributed to the higher ability of HPMC15LV to stabilize curcumin. The HPMC15LV-curcumin composite was about three-fold more cytotoxic to A549 lung cancer cells than free curcumin and was more potent in inducing apoptosis in cancer cells. These results demonstrate that the anti-cancer activity of curcumin encapsulated in a polymer composite is greater compared to that of the free drug. In another study, Liu et al. synthesized mPEG-b-PHEMA-5HA to load curcumin into micelles [75]. Curcumin and the polymer were cross-linked in the micelles using the phenol–yne click reaction. The cross-linked micelles were uniform in size and exhibited higher drug loading than the unlinked micelles. In addition, the cross-linked micelles were more stable when diluted with water than the unlinked micelles and showed increased release capability under acidic conditions (pH 5). The researchers speculated that cross-linking provides structural stability to the micelles and that a low pH facilitates the breakdown of the vinyl ester bonds. These cross-linked micelles were highly cytotoxic to cancer cells and effectively inhibited cancer cell growth in mice at high systemic concentrations. Shitole et al. reported on polymeric nanocapsule (NCS) that entrapped DTX and quercetin for targeting prostate cancer [76]. A chemically modified polymer was prepared by conjugating the luteinizing-hormone-releasing hormone (LHRH) ligand to poly-lactide-co-glycolide (PLGA) using PEG. The NCSs carrying the two drugs were uniform in size (120–140 nm) and had a negative surface charge. The drug release pattern was similar for both drugs; however, the release was sustained in the presence of the PEG coating. This suggests that PEG coating enhances the stability of DTX and quercetin in the serum. The NCSs effectively targeted cells expressing LHRH-recognizing receptors, thus, a significantly increased uptake was observed in cancer cells. Mechanistically, NCSs exhibited potent cytotoxicity by increasing the caspase 3 activity. It was also observed that LHRH-NCSs can accumulate in cancer cells in vivo, thereby effectively inhibiting cancer growth and differentiation.

Hydrophilic and hydrophobic polymers can be combined to form micelles. Ma et al. co-encapsulated DOX and curcumin in hyaluronic acid-vitamin E succinate (HA-VES) polymeric micelles (PMs) [77]. These PMs exhibited a uniform size of 223 nm and a high encapsulation efficiency for DOX (94%) and curcumin (72%). MCF-7/Adr cells are DOX-resistance cancer cells that overexpress P-glycoprotein (P-gp), a transporter that facilitates drug efflux. The cytotoxicity of these PMs in MCF-7/Adr cells was 14.83-fold higher than that of free DOX because curcumin can inhibit P-gp, and micelle encapsulation enhances the stability of curcumin. The internalization of PMs by cancer cells is energy-dependent and caveolae-mediated, an endocytic process facilitated by HA-CD44 interaction. Furthermore, these PMs induced apoptosis in MCF-7/Adr cells, which was due to increased cellular uptake and increased retention time. In vivo, DOX- and curcumin-encapsulated PMs had a longer half-life, increased retention time in the blood, and a higher plasma concentration compared to the free drugs. Co-delivery of polymeric micelles efficiently inhibited tumor growth in mice without any adverse effects.

A dendrimer is a nanoparticle with a branched polymer. Ben-Zichri et al. used dendritic polymer nanoparticles to co-formulate resveratrol and curcumin [78]. The two polyphenols wrapped in a dendrimer of size about 130 nm showed a significant reduction in light-induced decay, indicating the stability of the nanoparticles. The authors explained that resveratrol/curcumin dendrimer targets mitochondria to induce cytotoxicity in SH-SY5Y cancer cells. Mechanistically, these nanoparticles targeted the mitochondria, leading to an increase in intracellular calcium release, a decrease in cytochrome C oxidase activity, and depolarization of the mitochondrial membrane.

### 2.3. Protein Formulation

Proteins are natural polymers present in plants and animals that can be used as stable drug-delivery materials with low toxicity and biodegradability. The amphiphilic nature of proteins can improve solubility and stability regardless of the type of polyphenol. Furthermore, proteins can interact with polyphenols to form colloids or biocompatible nanoparticles with a high carrying capacity [79]. In addition, surface modification of protein nanoparticles can easily modify the functional groups or place additional protective layers for effective formulation [80]. Suktham et al. used sericin nanoparticles to deliver resveratrol [81]. The highly biocompatible protein particles had a size of 200–350 nm and an EE of 71–75%. Interestingly, the viability of CRL-2522 normal cells treated with high concentrations of resveratrol-loaded nanoparticles was higher compared to that of cells treated with free resveratrol because sericin enhances fibroblast adhesion and cell proliferation. In Caco-2 cancer cells, these nanoparticles exhibited significantly higher cytotoxicity, with 97% cellular uptake after 24 h of incubation. These results showed that resveratrol-loaded sericin nanoparticle formulation enhanced the anticancer effects of the polyphenol, and these nanoparticles were non-toxic to normal cells and even supported their growth.

Protein-based formulations can also be coated for specific purposes and protection. Zhang et al. fabricated surface-modified protein nanoparticles that can cross the BBB to target glioblastoma [82]. The zein protein–curcumin colloid was coated with polydopamine to increase its stability and functionalized with a dodecamer peptide (G23) to enhance BBB permeability and cancer tissue penetration. These nanoparticles were about 120 nm in size and had a negative surface charge. G23 functionalization increased the basolateral accumulation of the nanoparticles near the BBB two-fold, which improved their ability to penetrate the BBB. Furthermore, these nanoparticles exhibited a high ability (64%) to penetrate cancer cell spheroids, suggesting that these particles can target cancer cells within the brain. Compared to free curcumin, the curcumin nanoparticles exhibited increased cytotoxicity and were able to decrease the proliferation (two-fold) and migration (50%) of C6 glioma cancer cells. Thus, the functionalized zein nanoparticles not only increased the stability of curcumin but also promoted effective drug delivery to cancer cells within the brain.

Some proteins capable of self-assembly are widely used in drug delivery research. Han et al. formulated curcumin using hydrophobin HGFI. HGFI was fused with the GE11 peptide to target the EGFR, which is highly expressed in some types of cancers [83]. The amphiphilic HGFI increased the solubility of curcumin and exhibited a high EE (88%). In addition, the EGFR-targeting ability of GE11 improved the uptake of nanoparticles in EGFR-overexpressing HCT 116 cancer cells approximately two-fold. Furthermore, these nanoparticles showed high cytotoxicity and specificity even at concentrations where free curcumin showed no toxicity. Lu et al. used amphiphilic zein protein to improve the low loading efficiency of regenerated silk protein when formulating hydrophobic paclitaxel and curcumin [84]. The generated nanospheres had a unique structure with a single hole and exhibited a size of 265 nm and an EE of 81% and 93% for paclitaxel and curcumin, respectively. Furthermore, paclitaxel/curcumin nanospheres greatly improved the cytotoxicity of each drug, reducing their IC50 value more than six-fold. In addition, the authors showed that paclitaxel and curcumin act synergistically on cancer cells, since the combined cytotoxicity was dependent on the ratio of the loaded drugs.

Razi et al. developed caseinate–chitosan nanoparticle-stabilized genipin for curcumin delivery [85]. Casein and chitosan electrostatically interacted to form particles, which were then cross-linked with genipin. The genipin-stabilized caseinate–chitosan nanoparticles (G-CCNPs) had a high EE of 88% due to the hydrophobic interaction between curcumin and caseinate. Unlike nanoparticles without cross-linking, G-CCNPs exhibited no variation in size and PDI value by pH, although the drug release was higher at pH 5.5 than at pH 7.4. These results showed that G-CCNPs were a stable delivery system to target the tumor microenvironment. Curcumin-loaded G-CCNPs significantly reduced the viability of HeLa cells, and the IC50 values for free curcumin and curcumin-loaded G-CCNPs were found to be 10 µg/mL and 6.5 µg/mL, respectively. The authors showed that these nanoparticles interact with the cell membrane, as imaging results demonstrated that G-CCNPs localize on the cell surface and are not internalized. Nevertheless, the study confirmed that G-CCNPs interact strongly with the cell surface and prevent the degradation of curcumin.

### 2.4. Other Materials

**Figure 4:**
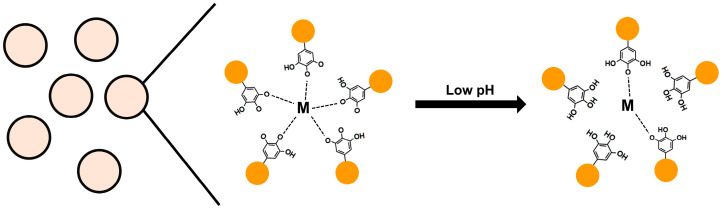
Metal phenolic network-based particle and pH-dependent property. M is metal ion.

Polyphenols act as antioxidants by chelating metal ions with their hydroxyl groups. Recently, the interaction between polyphenols and metal ions has been exploited to remove toxic metal ions and form nanoparticles or coatings for drug delivery [86]. The metal–phenolic network (MPN) exhibits different affinities depending on the number of hydroxyl groups and the type of metal ion and has a pH-dependent property where the binding force is weaker at lower pH [87,88] (Figure 4). This feature is being used to develop anticancer drugs because it enables controlled release by targeting the acidic environment of cancer [89]. Chen et al. developed curcumin nanoparticles using MPN between EGCG and Fe(III) ions [90]. Curcumin nanoparticles dispersed by MPN exhibited a high curcumin EE of 90% and a size of 180 nm. MPN formation is pH-dependent; at low pH, the interaction with metal ions is weakened and nanoparticles are disassembled due to the protonation of polyphenols. This phenomenon was confirmed by in vitro studies showing that the amount of curcumin released was 90% at pH 5 and 34% at pH 7.4. Thus, once inside the cancer cells, curcumin can be rapidly released within the endosome or lysosome due to its low pH. Curcumin nanoparticles exhibited significantly higher cytotoxicity against MCF-7 cancer cells, which was associated with Bcl-2 down-regulation and Bax and caspase 3 up-regulation-induced apoptosis. In addition, these nanoparticles inhibited the migration and invasion of cancer cells more effectively than free curcumin in vitro. These particles did not induce hemolysis and blood toxicity, and compared to free drug, tumor growth was inhibited nearly two-fold when treated with curcumin nanoparticles in mice bearing MCF-7 xenografts.

DNA is a double-stranded genetic material that contains the information necessary for life. A distinctive feature of DNA is hydrogen bonding between adenosine and thymine and between guanine and cytosine [91]. This feature makes it possible to systematically engineer DNA into specific structures, which has attracted interest in biomedical fields such as gene therapy, DNAzymes, and drug delivery [92]. Han et al. developed a complex that simultaneously delivers genes and polyphenols and exhibits anticancer effects [93]. The structure was developed based on the interaction between the polyphenol tannic acid and branched DNA. An aptamer was attached to the surface of the nanocomplex to target the cancer cells. In the presence of Mg2+ ions, DNAzymes can inhibit the migration of cancer cells by suppressing the expression of MMP-9. The self-assembled structure showed a high loading efficiency of tannic acid (85%) and therapeutic genes (95%). In addition, this structure enabled controlled release, depending on the stimulation in cancer cells. The nanocomplex can be disassembled to release tannic acid in a low pH environment, such as that present in endosomes.

The branched DNAs can be cleaved by glutathione and DNase I in the cytoplasm, releasing the DNA. In A549 lung cancer cells, these nanocomplexes with an aptamer had a seven-fold higher cellular uptake. Correspondingly, cell proliferation and migration were significantly inhibited. In addition, decreased levels of *C-raf* mRNA and MMP-9 protein confirmed that the polyphenol-DNA nanocomplex worked properly within cells. Finally, the authors confirmed that the nanocomplexes accumulate in the tumors in vivo due to the presence of the aptamer, thus effectively preventing cancer growth. In another study, tannic acid was used to form a nanocomplex of branched DNA/RNA and cancer cell membrane for chemotherapy [94]. The nucleic acid was used as a small interfering RNA (siRNA) to induce RNA interference (RNAi) of PLK1, which is overexpressed in cancer, using siPLK1 RNA and Y-shaped DNA to form nanostructures. In addition, A549 lung cancer cell membrane (A549m) was coated with tannic acid to induce homotypic targeting to A549 cells. The nanocomplexes with A549m had a size of 164.2 nm and a negative surface charge. The nucleic acid-tannic acid nanocomplexes degraded under acidic conditions, but the membrane coating stably protected the nanocomplexes until endosomal/lysosomal fusion in the target cells. In addition, the nanocomplexes with A549m showed a five-fold higher targeting ability than those without A549m, while macrophage uptake was reduced by 30%. In A549 cells, the nanocomplex with A549m reduced PLK1 expression and viability by approximately 60%. In addition, the tannic acid in the nanocomplex increased the rate of cancer cell apoptosis 20-fold. In the bloodstream, the nanocomplex with A549m had a half-life of 2.13 h, which was 26-fold and two-fold longer than siPLK1 and the nanocomplex, respectively. When injected into mice, the nanocomplex with A549m reduced PLK1 mRNA by 50% and tumor growth more than five-fold compared to the control group.

The delivery systems of polyphenols using polymers, proteins, and other materials mentioned above are summarized in Table 3, Table 4 and Table 5.

## 3. Consideration for Polyphenol Delivery System

Interest in polyphenolic drug delivery systems is growing due to enhanced therapeutic effects on drug-resistant cancer cells from improving drug resistance [95]. Long-term use of traditional chemotherapeutic agents can lead to drug resistance in cancer cells that proliferate and differentiate rapidly, such as through drug efflux or drug metabolism alteration [96]. Drug cocktails, which consist of drugs with different effects, are being used to address this problem [97,98]. In this regard, polyphenols are less toxic to normal cells than conventional chemotherapeutic agents [99] and can be regulated through various mechanisms [99,100]. Co-delivery of polyphenols with other anticancer drugs shows the possibility for more effective cancer treatment with lower side effects on normal cells and synergistic effects [98] (Table 6). For example, P-glycoprotein, involved in drug efflux, is a membrane transporter that uses ATP to transport drugs out of the cell and is one of the main contributors to drug resistance [101]. Studies have shown that various polyphenols, including EGCG [102], flavonoids [103], and curcumin [104], inhibit the activity and expression of p-glycoprotein, thereby enhancing the effectiveness of chemotherapeutic agents when combined. Polyphenols also help to treat cancer by inducing programmed cell death [95]. In particular, autophagy is essential for maintaining cellular homeostasis, and it plays a role in suppressing tumor development by allowing cells to remove damage caused by oxidative stress or inflammation [105]. Inducing autophagy-associated programmed cell death has received increasing attention as a way to treat cancer [106]. Several studies have demonstrated that polyphenols can enhance autophagy by activating autophagy-related signaling pathways, such as the AMPK/mTOR and PI3K/Akt/mTOR pathways [107,108]. Modulation of autophagy-related signaling by polyphenols can induce cell death in drug-resistant cancer cells and increase the sensitivity of drugs, thereby enhancing therapeutic effects [109].

Although much research has been conducted on using polyphenols as anticancer agents and positive results have been accumulated, their application in practice is still limited. The main reasons for this are their low bioavailability [45] and multitargeting [114], which reduces their effectiveness. The low solubility and stability of most polyphenols make it difficult for them to be effective in the body [115]. Furthermore, as multi-targeting agents, polyphenols can inhibit multiple signaling pathways involved in cancer cell survival, but they can also cause various side effects in the body. For example, their Fe-chelating ability can reduce Fe, which induces oxidative stress; on the other hand, it can also lead to a deficiency of Fe in the body [114]. Moreover, genistein, a phytoestrogen, can disrupt hormone communication. Polyphenols, like other drugs, require specific and reliable targeted delivery to overcome these issues [116]. Despite the potential therapeutic benefits of polyphenols, several DDSs are being developed by applying different biocompatible materials to overcome their practical limitations. Furthermore, depending on the location and type of target in the body, materials with modifications or appropriate properties for targeted and controlled delivery should be selected and fabricated [117]. Although many results show dramatic anticancer effects of polyphenols through formulation, such as the studies described in Section 2, applying nanoformulation as a DDS is rare. One of the concerns is that the toxicity of the delivered drug does not translate to the nanoformulation, which means that sufficient toxicity studies are required [118]. In addition, the main limitation of nanoparticles is that they are rapidly eliminated due to their size, even though they have increased retention time and penetration in the blood compared to free drugs. Nanosized particles are generally selected for a size of 30–200 nm to avoid renal clearance or elimination by macrophages, and it is assumed that an EPR effect can be expected [119,120]. However, interactions with other substances in the blood can also aid elimination. Furthermore, their application in oral administration, the most accessible consumer route, remains challenging. Degradation by acidic conditions in the stomach, penetration or passage through intestinal endothelial cells, pH changes due to the different intestinal environment depending on the patient’s lifestyle, and the distribution and interaction of intestinal microorganisms may alter the effectiveness [120,121,122]. Therefore, for applying nanoformulation, research should be continuously conducted to consider and reflect pharmacokinetics and the body environment.

## 4. Conclusions

Among agents discovered and developed to treat cancer, polyphenols are attractive compounds. Polyphenols present in plant extracts naturally possess antioxidant activities against cancer cells. These bioactive compounds can modulate the inflammatory response besides acting as scavengers of ROS. Targeting pathways of cancer development and aggravation has gained importance, and recent studies have used polyphenols as potent agents to inhibit cancer progression. However, most polyphenolic compounds have rarely been used in clinical trials due to their instability, poor water solubility, and poor bioavailability.

DDS offers significant advantages and can be used to improve the bioavailability and therapeutic efficacy of polyphenols. Encapsulation can protect polyphenols from degradation and can improve their solubility and absorption in the body. DDS can also help target specific tissues or cells and regulate the release over time, providing sustained therapeutic effects. These benefits can help unlock the full therapeutic potential of polyphenols and improve health outcomes.

Although the enhancement effects of DDS have been focused on inhibiting cancer growth, proliferation, and metastasis, there is a paucity of studies confirming the direct impact of these systems on amplifying the antioxidant and anti-inflammatory effects of polyphenols. The antioxidant, anti-inflammatory, and anti-cancer effects of polyphenols have been well established. Therefore, it is reasonable to expect that the use of DDSs will have a synergistic effect. DDS with polyphenols can be more effective with optimizing formulations and understanding their precise mechanisms. These will help to ensure reliable targeted delivery and controlled release, which are essential factors in the successful use of polyphenols as therapeutics to inhibit cancer progression.

## Figures and Tables

**Table 1:** Methods and properties of lipid-based system.

Materials	Polyphenols	Methods and Functionalization	EE * and LC **	Particle Size	Ref.
Liposome	Resveratrol	PEGylation TF moiety	EE ~75%	211.2 nm	[55]
		Peptide liposome	EE > 90%	140 nm	[56]
	Curcumin	Co delivery with gefitinib PEGylation	EE > 80%	110–130 nm	[57]
Phytosome	Genistein	With MCT or LCT Solvent evaporation method	EE > 95%	150–300 nm	[61]
	Thymoquinone	Soy-phospholipid	No data	45.6 nm	[62]
	Curcumin	PEGylation Folate modification	LC 24.3%	185.3 nm	[63]
	*Moringa oleifera* leaf polyphenols	Soy-phosphatidylcholine Thin-layer hydration method	EE 90.23%	296 nm	[64]
Nanostructured lipid particle	Curcumin	Cinnamon oil, Tween 80 PEG 200, Absorption enhancer	No data	106 nm	[66]
	Cornus mas extract (CME)	Cubosome Glyceryl monooleate Poloxamer^®^ 407 Eudragit^®^ S100	EE 95.14% LC 9.51%	22.7 nm	[67]
	EGCG	Arginyl-glycyl-aspartic acid (RGD) modification	EE 80% LC 75%	85 nm	[68]
	Apigenin	Hyaluronic acid modification Co-treatment with docetaxel	EE 70% LC 3.5%	88 nm	[69]

* EE: Encapsulation efficiency % (loaded polyphenol amount/initial polyphenol amount × 100); ** LC: Loading capacity % (Loaded polyphenol weight/polyphenol-loaded particle weight × 100).

**Table 2:** Improvement effects of polyphenols using lipid-based system.

Materials	Polyphenols	Drug Release	Target	Improvement	Ref.
Liposome	Resveratrol	Slow release (No data)	U-87 MG human glioblastoma cells	Apoptosis 2.5-fold ↑ In vivo tumor volume 2-fold ↓	[55]
		98% at pH 5.5 30% at pH 6.8 (~48 h)	MCF-7 breast cancer cell	Cytotoxicity 26% ↑ Apoptosis induced p53, Bax, Bcl-2, and caspase	[56]
	Curcumin	At pH 7.4 (~48 h) >90% (alone) >70% (with gefitinib)	PC-9 Gefitinib-resistant H1975	Cytotoxicity 10–15% ↑ In vivo tumor growth inhibition 45% ↑	[57]
Phytosome	Genistein	Unstable in pH 4.5	Hepatocellular carcinoma	Liver toxicity reduction (AST 30%, ALT 10%) Caspase 8 ↑ Angiogenesis and invasion↓	[61]
	Thymoquinone	>95% (45%↑) (~12 h)	A549 Human lung cancer	IC50 < 5 μM (4-fold ↓) Caspase 3 expression ↑	[62]
	Curcumin	50% at pH 5.5 20% at pH 7.4 (~48 h)	HeLa cell	IC50 11.9 μg/mL (3-fold ↓) Selective accumulation in cancer 40% ↑	[63]
	*Moringa oleifera* leaf polyphenols	At pH 7.4 43% (~8 h) 53% (~72 h)	4T1 cancer cell	Stability in gastric condition 2-fold ↑ Cytotoxicity 4-fold ↑ Proliferation inhibition (more than DOX at 100 μg/mL)	[64]
Nanostructured lipid particle	Curcumin	80% at pH 1.2 >95% at pH 4.6 (~24 h)	Caco-2 Human colon cancer	Cytotoxicity 57% ↑ C_max_ 5-fold ↑ AUC 4.5-fold ↑	[66]
	*Cornus mas* extract (CME)	Sustained (~80 h) >90% intestinal >70% gastric	Caco-2 Human colon cancer	DPPH scavenging 27%↑ Cytotoxicity DNA fragmentation↑ Cell cycle arrest (G0-G1 phase) ↑	[67]
	EGCG	No data	MDA-MB-231 human breast cancer	IC50 38.8 μM (22.5% ↓) Cell cycle arrest Apoptosis ↑	[68]
	Apigenin	Sustained (~80 h) 90% at pH 5.5 85% at pH 7.4	A549 Human lung cancer	IC50 23.51μM (3-fold ↓) Apoptosis 15% ↑	[69]

↑: Increase in some effects; ↓: Decrease in some effects.

**Table 3:** Methods and properties of nanoformulation using polymer, protein, or other materials.

Materials	Polyphenols	Methods and Functionalization	EE * and LC **	Particle Size	Ref.
Polymer	Hesperestin	Chitosan-folate conjugate Sodium tripolyphosphate (cross-linking)	EE 98%	457 nm	[72]
	Quercetin	Eudragit S100	EE 41.8% LC 2.2%	66.8 nm	[73]
	Curcumin	Spray drying HPMC15LV or PVP-K30 Lactose monohydrate	EE 13~78%	4.8–8.3 nm	[74]
		Phenol–yne click reaction mPEG-b-PHEMA-5HA	EE 43.4% LC 17.8%	104 nm	[75]
	Quercetin	Luteinizing-hormone-releasing hormone (LHRH) ligand PLGA, PEG Co-delivery with docetaxel	EE 75.8%	120–140 nm	[76]
	Curcumin	Co-delivery with doxorubicin Polymeric micelle Hyaluronic acid–vitamin E succinate	EE 72% LC 8.3%	223 nm	[77]
	Resveratrol and curcumin	Bolton W3000 dendritic polymer solvent displacement method	No data	130 nm	[78]
Protein	Resveratrol	Sericin silk protein	EE 71–75%	200–350 nm	[81]
	Curcumin	Zein protein Dodecamer peptide (G23) Polydopamine	EE 81.7% LC 8.11%	106.3 nm	[82]
		Hydrophobin (HGFI) GE11 peptide	EE 88% LC 47%	80 nm	[83]
		Zein protein Silk protein Co-formulation with Paclitaxel	EE 93% LC 12%	265 nm	[84]
		Caseinate–chitosan Genipin cross-linking	EE 88.6% LC 4.2%	272 nm	[85]
Iron ion	Curcumin	Metal–phenolic network between EGCG and iron, coating	EE 90% LC 40%	184.8 nm	[90]
Nucleic acid	Tannic acid	Branched DNA, Aptamer antisense of *C-raf* mRNA	EE 85%	150 nm	[93]
		Branched DNA, siPLK1 RNA A549 membrane	No data	164.2 nm	[94]

* EE: Encapsulation efficiency % (loaded polyphenol amount/initial polyphenol amount × 100); ** LC: Loading capacity % (Loaded polyphenol weight/polyphenol-loaded particle weight × 100).

**Table 4:** Improvement effects of polyphenol nanoformulation using polymer.

Materials	Polyphenols	Drug Release	Target	Improvement	Ref.
Polymer	Hesperestin	~45% at pH 7.4 <20% at pH 3 (~72 h)	HCT15 Human colon cancer cell	IC50 28 μM (6.7-fold↓) Apoptosis 2.4-fold↑ Bax and Bad mRNA 35%↑	[72]
	Quercetin	No release at < pH 4.5 (~2 h) 90% at pH 7.4 (~20 h)	CT26 colon cancer cell	IC50 0.8 μM (81-fold↓) Cell viability 40%↓	[73]
	Curcumin	~28% at pH 7.4 (~90 min)	A549 Human lung cancer	Solubility 14-fold↑ IC50 44 µM (3-fold↓)	[74]
		~25% at pH 5 ~72% at pH 7.4 (~30 h)	4T1 cancer cell HeLa cell	IC50 4–9 µg/mL Half-life 6.16 h (14-fold↑) Tumor growth 27%↓	[75]
	Quercetin	95% at pH 7.4 (with serum, ~48 h)	PCa cell lines (PC-3 and LNCaP)	IC50 36–82%↓ Caspase 3 activation↑ in vivo tumor growth 40%↓	[76]
	Curcumin	~69% at pH 4.5 ~37% at pH 7.4 (~24 h)	MCF-7 cell MCF-7/Adr cell	Reduction of drug efflux IC50 14.8-fold↓ Apoptosis 4.6-fold↑ Tumor inhibition 55.2% (2-fold↑)	[77]
	Resveratrol and curcumin	Earlier in pH 5.4 than pH 7	SH-SY5Y cancer cell	Cytotoxicity 37%↑ Intracellular calcium release 20%↑ Cytochrome C oxidase activation↓ Mitochondrial depolarization	[78]

↑: Increase in some effects; ↓: Decrease in some effects.

**Table 5:** Improvement effects of polyphenol nanoformulation using protein or other materials.

Materials	Polyphenols	Drug Release	Target	Improvement	Ref.
Protein	Resveratrol	65% at pH 7.4 (~72 h, high concentration)	Caco-2 cancer cell	97% cellular uptake after 24 h IC50 < 6% wt No toxicity to normal cell (100% wt)	[81]
	Curcumin	80% at pH 5 45% at pH 7.4 (~48 h)	C6 glioma cell	Blood brain barrier penetration↑ (2-fold↑) Cancer proliferation and migration > 50%↓	[82]
		At pH 7.4 ~53% (~8 h) ~94% (~72 h)	HCT 116 cancer cell	Cellular uptake 2-fold↑ IC50 3.7 µg/mL (free curcumin is not toxic to 20 µg/mL)	[83]
		Sustained~180 h ~66% at pH 5 ~49% at pH 7.4	MCF-7 breast cancer cell	IC50 0.72 µg/mL (6-fold↓) Synergistic effect (CI > 1)	[84]
		77% at pH 5.5 60% at pH 7.4 (~6 h)	HeLa cell A549 cell	IC50 6.5 µg/mL (35%↓) Cell surface attachment and localization	[85]
Iron ion	Curcumin	91% at pH 5 34% at pH 7.4 (~24 h)	MCF-7 human breast cancer cell	Uptake efficiency 93% after 3 h Bcl-2 down-regulation (2.6-fold↓) Bax and caspase 3 up-regulation (40%↑)	[90]
Nucleic acid	Tannic acid	Disassembly in pH 5.5	A549 human lung cancer cell	Cellular uptake 7-fold↑ Apoptosis 40%↑ by tannic acid and 20%↑ by therapeutic genes	[93]
		In acidic pH (when fused with endosomal/lysosomal vesicle)	A549 human lung cancer cell	Homotypic targeting (3-fold↑) Macrophage uptake 30%↓ Half-life 2.13 h (26-fold↑) Tumor growth 5-fold↓	[94]

↑: Increase in some effects; ↓: Decrease in some effects.

**Table 6:** Synergistic effects in cancer therapy using combination of polyphenols with other agents.

Combination	DDS Method	Target/Treated Conc.	Results	Ref.
Erlotinib and resveratrol	Nanostructured lipid particle Hot homogenization method Miglyol and Precirol Poloxamer 470 Drug ratio 1:8	A549 human lung cancer cell /E 5 µg/mL and R 40 µg/mL	Cell viability 12.6% (75%↓) Apoptosis 85.5% (20–30%↑) (Bax and p53 expression↑, Caspase 3, 8, and 9 activation↑) Cell cycle arrest 40% in G2/M phase	[110]
Docetaxel and resveratrol	PEGylated nano-liposomes Thin-film hydration method Soy lecithin, Cholesterol, and DSPE-MPEG2000 Drug ratio 1:2	PC3 and DU145 Human prostate cancer cell lines/D 32.3 µg/mL and R 18.3 µg/mL PC3-bearing mice/ D 10 mg/kg and R 5.65 µg/mL	IC50 (docetaxel) 1.49 µg/mL (10-fold↓) Apoptosis induction 69% (2-fold↑) Cellular uptake 3.5–5.5-fold↑ In vivo tumor growth inhibition (Volume 3-fold↓) Survival day ~42 day, (5-fold↑ than free drugs) In vivo toxicity reduction	[111]
Cisplatin and curcumin	mPEG urethane gemini surfactant nanoparticle	MDA-MB-231 breast cancer cells/ Ci 13 µM and Cu 20 µM	Sub G1 cell ratio 20% (2–4-fold↑) Late apoptosis 59% (39–290%↑) Bax/Bcl-2 ratio 50–200%↑	[112]
Doxorubicin and quercetin	Polymeric nanoparticle Biotin-PEG_2k_-PCL_5k_	MCF-7 and MCF-7/ADR breast cancer cells /D 5 μg/mL and Q 11.75 μg/mL MCF-7/ADR-bearing nude mice/D 5 mg/kg and Q 11.75 mg/kg	Drug resistance↓(136-fold↓) Drug efflux 15–48%↓ P-glycoprotein activity 1.8–2.5-fold↓ In vivo tumor volume 3-fold↓	[113]

↑: Increase in some effects; ↓: Decrease in some effects.

## Data Availability

Not applicable.
